# Specific Marking of hESCs-Derived Hematopoietic Lineage by *WAS*-Promoter Driven Lentiviral Vectors

**DOI:** 10.1371/journal.pone.0039091

**Published:** 2012-06-14

**Authors:** Pilar Muñoz, Miguel G. Toscano, Pedro J. Real, Karim Benabdellah, Marién Cobo, Clara Bueno, Verónica Ramos-Mejía, Pablo Menendez, Per Anderson, Francisco Martín

**Affiliations:** 1 Human DNA Variability Department, Pfizer-Universidad de Granada-Junta de Andalucía Centre for Genomics and Oncological Research (GENYO), Granada, Spain; 2 Oncology Department, Pfizer-Universidad de Granada-Junta de Andalucía Centre for Genomics and Oncological Research (GENYO), Granada, Spain; French Blood Institute, France

## Abstract

Genetic manipulation of human embryonic stem cells (hESCs) is instrumental for tracing lineage commitment and to studying human development. Here we used hematopoietic-specific Wiskott-Aldrich syndrome gene (*WAS*)-promoter driven lentiviral vectors (LVs) to achieve highly specific gene expression in hESCs-derived hematopoietic cells. We first demonstrated that endogenous *WAS* gene was not expressed in undifferentiated hESCs but was evident in hemogenic progenitors (CD45**^−^**CD31^+^CD34^+^) and hematopoietic cells (CD45^+^). Accordingly, *WAS*-promoter driven LVs were unable to express the *eGFP* transgene in undifferentiated hESCs. eGFP^+^ cells only appeared after embryoid body (EB) hematopoietic differentiation. The phenotypic analysis of the eGFP^+^ cells showed marking of different subpopulations at different days of differentiation. At days 10–15, AWE LVs tag hemogenic and hematopoietic progenitors cells (CD45**^−^**CD31^+^CD34^dim^ and CD45^+^CD31^+^CD34^dim^) emerging from hESCs and at day 22 its expression became restricted to mature hematopoietic cells (CD45^+^CD33^+^). Surprisingly, at day 10 of differentiation, the AWE vector also marked CD45**^−^**CD31^low/−^CD34**^−^** cells, a population that disappeared at later stages of differentiation. We showed that the eGFP^+^CD45**^−^**CD31^+^ population generate 5 times more CD45^+^ cells than the eGFP**^−^**CD45**^−^**CD31^+^ indicating that the AWE vector was identifying a subpopulation inside the CD45**^−^**CD31^+^ cells with higher hemogenic capacity. We also showed generation of CD45^+^ cells from the eGFP^+^CD45**^−^**CD31^low/−^CD34**^−^** population but not from the eGFP**^−^**CD45**^−^**CD31^low/−^CD34**^−^** cells. This is, to our knowledge, the first report of a gene transfer vector which specifically labels hemogenic progenitors and hematopoietic cells emerging from hESCs. We propose the use of *WAS*-promoter driven LVs as a novel tool to studying human hematopoietic development.

## Introduction

Human embryonic stem cells (hESCs) are derived from the inner cell mass of preimplantation embryos. They can differentiate into cell types of the three germinal layers and under specific culture conditions proliferate indefinitely [1], [2], [3]. hESCs represent a unique tool for studying early human development, for cell therapy and for developing *in vitro* models of human diseases [4], [5], [6]. Genetic modification of hESCs is fundamental to explore the mechanisms governing the balance between self-renewal and lineage commitment through overexpression or silencing of specific genes [7]. In addition, tracing lineage specification demands the ability to express a reporter/marker gene (i.e. *eGFP*) exclusively when lineage-specific cells emerge *in vitro*/*in vivo*. However, unlike their murine counterparts, genetic modification of hESCs is still very challenging due to the low efficiency of existing delivery methods and to the strong silencing of the transgenes [8].

Lentiviral vectors (LVs) are a powerful integrative system [9], [10] that can transduce most cell types including hESCs [11], [12]. They are one of the most promising gene delivery methods for hESCs due to their high transduction efficiency and stable expression. Latest generation self-inactivated lentiviral vectors (SIN-LVs) express the transgene under internal promoters [13], [14] allowing the use of physiological or tissue-specific promoters to obtain regulated transgene expression [15]. Using this technology, several groups have developed cardiac-specific lentiviral vectors able to specifically mark cardiomyocites derived from hESCs [16], [17], [18]. However, no LVs capable of specifically marking blood cells differentiating from hESCs have been reported so far.

*In vitro* differentiation of hESCs toward the hematopoietic lineage provides a unique tool not only to study human hematopoietic development and as a platform for drug screening but also as a potential source for cell-gene therapy strategies [19], [20], [21], [22], [23]. Using the embryoid body (EB) differentiation model [24], [25], hESC-derived hematopoietic cells emerge from a subset of hemogenic progenitors expressing CD31, CD34, but lacking CD45 (CD45**^−^**CD31^+^CD34^+^ hemogenic progenitors) [26]. Based on the CD34 expression levels, the CD45**^−^**CD31^+^ cells can be differentiated into hemato-endothelial progenitors (CD45**^−^**CD31^+^CD34^bright^)(also positive for VE-Cadherin and KDR) and the hematopoietic-restricted progenitors (CD45**^−^**CD31^+^CD34^dim^) [4], [27]. However, despite the fact that hESC-derived hematopoietic cells show colony-forming unit (CFU) capacity and a phenotype similar to somatic hematopoietic cells, the *in vivo* generation of fully functional hESC-derived HSCs capable of engrafting immunodeficient recipients still remains a challenge [21], [28], [29], [30] and will likely depend upon further understanding of intrinsic molecular determinants.

Targeted expression of genes in hESCs-derived hematopoietic cells will help to elucidate the mechanisms governing early hematopoietic development and to design more efficient strategies for the generation of hematopoietic stem cells (HSCs) from hESCs. Our group has previously developed two different hematopoietic-specific LVs, WE [31], [32] and AWE [33] driving the expression of eGFP through different promoter fragments of the Wiskott-Aldrich Syndrome (WAS*)* gene (*WAS*). The *WAS* gene codifies a hematopoietic specific protein involved in translating extracellular signals to actin cytoskeleton polymerization and its expression is driven by two different promoters, the proximal promoter [34] and the alternative promoter located 3 kb upstream [35]. The WE vector contains a 500 bp fragment of the proximal promoter and the AWE vector harbors a longer version containing an additional 387 bp fragment of the alternative *WAS* promoter. In the present study we demonstrated the usefulness of both the WE and AWE LVs in achieving highly specific transgene expression in hESCs-derived hemogenic progenitors and hematopoietic cells. eGFP expression in WE- and AWE-transduced hESCs paralleled the expression of endogenous *WAS mRNA*. Indeed, both LVs achieved efficient integration in undifferentiated hESCs but they were unable to express the transgene. However, *eGFP* expression was efficiently driven by both vectors upon hematopoietic directed differentiation. At day22 of hematopoietic differentiation most eGFP^+^ cells were mature hematopoietic cells (CD45^+^CD33^+^). However, at days 10–15, the *WAS*-promoter driven LVs (WE and AWE) tag cells with a phenotype corresponding to hematopoietic-restricted hemogenic progenitors (CD45**^−^**CD31^+^CD34^dim^) and hematopoietic progenitors (CD45^+^CD31^+^CD34^dim^). Interestingly, at day 10 of differentiation, these vectors also marked CD45**^−^**CD31^low/−^CD34**^−^** cells. In vitro assays of sorted populations showed hemogenic potential of eGFP^+^CD45**^−^**CD31^+^ and eGFP^+^CD45^+^CD31^+^ as well as eGFP^+^CD45**^−^**CD31^low/−^CD34**^−^** cells. Therefore, the AWE vectors were able to identify a new population derived from hESCs with hemogenic potential. To our knowledge, this is the first report of a gene transfer vector which specifically tags hemogenic progenitors and hematopoietic cells emerging from hESCs. This data opens up new avenues for tracing human embryonic hematopoietic commitment allowing not only the isolation, purification and characterization of early hematopoietic progenitors but also the study of master hematopoietic genes.

## Methods

### Cell Lines and Culture Media

293T cells (CRL11268; American Type Culture Collection; Rockville, MD) were grown in Dulbecco’s Modified Eagle Medium (DMEM, Invitrogen, Edinburgh, Scotland) with GlutaMAX™ and supplemented with 10% Fetal Bovine Serum (FBS) (PAA Laboratories GmbH, Austria). Raji cells (CCL-86; ATCC) were grown in RPMI-1640 media (Gibco) supplemented with 10% FBS and glutamine 20 mM. Primary T cell lines (AlloT) were generated and mantainned in our laboratory by weekly allo-stimulation with mitomycin-C-treated Raji B-cells and cultured in RPMI-1640 media supplemented with 10% FBS and glutamine 20 mM. HUVEC cells (TCS Cellworks, Buckingham, UK) were grown in Medium 199 (Invitrogen) and supplemented with 20% FBS, endothelial growth supplements (ECGS, Sigma Aldrich), glutamine and heparin, AND-1 (Spanish Stem Cell Bank. www.isciii.es) [36], SHEF-2 (UK Stem Cell Bank, Hertfordshire) and H9 (Wicell Research Institute Inc. Madison, WI) hESC lines were maintained undifferentiated in a feeder-free culture as previously described [37] in Matrigel (BD Biosciences, Bedford, MA)-coated p12 plates for lentiviral transduction and expanded in matrigel-coated T25 flasks. Human ESCs were fed daily with human mesenchymal stem cell-conditioned medium (MSC-CM) supplemented with 8 ng/ml bFGF (Miltenyi Biotech, Bergish Gladbach, Germany), as described [22]. Media was changed daily and the cells were split weekly by dissociation with 200 U/ml collagenase IV (Invitrogen). Approval from the Spanish National Embryo Ethical Committee was obtained to work with hESCs.

### *In vitro* Hematopoietic Differentiation through Embryoid Body (EB) Formation

Near confluent transduced hESCs (day 0) were treated with collagenase IV for 1 min, and scraped off from the matrigel. The hESCs were transferred to low-attachment plates (Corning Life Sciences, Amsterdam, The Netherland) and incubated overnight in media composed by KO-Dulbeccós modified Eaglés medium (Invitrogen) supplemented with 20% non-heat-inactivated FBS for hESCs (Gibco), 1 mM glutamine, 0.1 mM non-essential amino acids and 0.1 mM β-mercaptoethanol. The next day the EBs were centrifuged and the media was changed for the same media supplemented with BMP-4 (25 ng/ml), Flt-3L (300 ng/ml), SCF (300 ng/ml), IL-3 (10 ng/ml), IL-6 (10 ng/ml) and G-CSF (50 ng/ml) [Chadwick, 2003 #1330], with media changes every 4 days. EBs were harvested for mRNA extraction at days 0, 1, 3, 5, 7, 11, 15 and 22, and dissociated using collagenase B (Roche Diagnostic, Basel, Switzerland) for 2 hours at 37°C followed by 10 minutes incubation at 37°C with Cell Dissociation Buffer (Gibco, Billings, MT) for FACS analysis and Colony Forming Units (CFUs) assays.

### FACS Analysis and Colony Forming Units (CFUs) Assays

The cells were resuspended in Iscove’s Modified Dulbecco’s Media (IMDM) (for CFUs assays) or PBS1x +3%FBS+2 mM EDTA buffer (for FACS analysis). The cell suspension was filtered through a 70-µm cell strainer (BD Biosciences, Bedford, MA) and stained with fluorochrome conjugated monoclonal antibodies anti-CD31-PE, anti-CD33-PE, anti-CD34-PE-Cy7 antibodies (all from BD Biosciences) and anti-CD45-APC (Miltenyi Biotech, Bergish Gladbach, Germany). Live cells identified by 7-AAD viability dye exclusion were analyzed for surface-marker expression and eGFP expression using a FACS Canto II flow cytometer equipped with the FACS Diva analysis software (BD Biosciences). The populations analyzed were hemogenic progenitors (CD45**^−^**CD31^+^CD34^+^), hematopoietic progenitors (CD45^+^CD34^+^), myeloid cells (CD45^+^CD33^+^) and total blood cells (CD45^+^). For pluripotency markers expression of the transduced cells they were stained with the antibodies SEEA-3, SSEA-4, TRA-1-60, TRA-1-81 and OCT3/4 (BD Biosciences).

For CFU assays, 20.000–35.000 cells were filtered through a 40-µm cell strainer (BD Biosciences, Bedford, MA) and plated in methylcellulose H4434 (Stem Cell Technologies, Vancouver, Canada) supplemented with 30 U/ml of EPO. Cells were incubated at 37°C, 5% CO_2_ humidified atmosphere. The colonies were count based on morphological characteristics after 10 to 14 days [22] and analysed by FACS for CD14 (Miltenyi), CD45 and CD33 expression.

### Analysis of the Hemogenic Potential of eGFP+ Cells

AWE-transduced cells were induced towards haematopoiesis as described previously. At day 10 of differentiation, the EBs were dissociated with colagenase B, stained for CD31 and CD45 and washed with sterile PBS buffer supplemented with 2 mM EDTA, 3% FBS and penicilin/streptomycine. Live cells were identified by 7-AAD viability dye exclusion and four populations of cells were sorted with a FACSAria flow citometer (BD Biosciences): CD45**^−^**CD31^+^ GFP**^−^**, CD45**^−^**CD31^+^ GFP^+^, CD45**^−^**CD31**^−^** GFP**^−^** and CD45**^−^**CD31**^−^** GFP^+^. The sorted cells (7–130×10^3^) were cultivated in StemSpan media (StemCell Technologies Vancouver, Canada) supplemented with Flt-3L (300 ng/ml), SCF (300 ng/ml), IL-3 (10 ng/ml), IL-6 (10 ng/ml) and G-CSF (50 ng/ml), or in methylcellulose H4434. 7–15 days later, cells were analyzed by FACS for the generation of hematopoietic cells (CD45 expression) and colony formation.

### Lentiviral Vectors (LVs)

The WE LV carries a 500-bp fragment of the WAS proximal promoter driving the expression of eGFP [31], [32], [38] The AWE LV was engineered by inserting a 387-bp fragment of the *WAS* alternative promoter immediately upstream of the 500-bp *WAS* proximal promoter in the WE vector.^35^ Both vectors share the self inactivated (SIN) lentiviral backbone described by Zuffery et. Al [13]. The pLVTHM [39] (obtained from Addgene; plasmid 12247; http://www.addgene.org/12247) and CE [40] LVs, driving the expression of eGFP through the EF1-α and CMV promoters, respectively, were used as controls. All recombinant DNA research follows the National Institutes of Health guidelines.

### Vector Production and Transduction of hESCs

The human immunodeficiency virus (HIV) packaging (pCMVΔR8.91) and VSV-G (pMD2.G) plasmids (http://www.addgene.org/Didier_Trono) are described elsewhere [13], [41]. Vector production was performed as previously described [10]. Briefly, 293T cells were plated on amine-10-cm tissue culture grade Petri dishes (Sarstedt, Newton, NC) the day before transfection to ensure exponential growth and over 80% confluence [32]. The vector (WE, AWE, CE or pLVTHM), the packaging (pCMVΔR8.91) and envelope plasmids (pMD2.G) (proportion 3∶2∶1) were resuspended in 1.5 ml of Opti-MEM (Gibco) mixed with 60 µl of Lipofectamine 2000 (Invitrogen). The plasmid-lipofectamine mixture was added to pre-washed cells and then incubated for 6–8 h. After 48 h, viral supernatants were collected, filtered through 0.45 µm filter (Nalgene, Rochester, NY) and concentrated by ultracentrifugation (Beckman Coulter) or by ultrafiltration at 2000×g and 4°C, using 100 Kd centrifugal filter devices (Amicon Ultra-15, Millipore, Billerica, MA) as previously described [42]. For transduction, human ESCs were dissociated with collagenase type IV, scraped off of the matrigel and plated on fresh matrigel-coated-p12-well plates. Immediately, fresh viral particles were transferred onto them. The media was changed after 5 hours. When the colonies reached confluence they were split and expanded. The vector genome per cell of transduced-hESCs was calculated from genomic DNA of 100,000 transduced hESCs and 10-fold increasing amounts of plasmid DNA (from 10^2^ to 10^7^ copies) to determine the standard curve. The Q-PCR (Mx3005P, Stratagene, La Jolla, CA) reaction consisted of 40 cycles at 94°C (15 sec), followed by 60°C (30 sec) and 72°C (30 sec). eGFP primers used were: forward: 5′- GTTCATCTGCACCACCGGCAAG-3′ and reverse 5′- TTCGGGCATGGCGGACTTGA-3′.

### RNA Isolation, RT-PCR and Q-PCR Analysis

RNA from undifferentiated hESCs, hEBs, hemogenic precursors and CD45+ blood cells was isolated using TRIzol (Invitrogen). cDNA was generated with the SuperScript First-Strand Synthesis System for RT-PCR (Invitrogen) and High Capacity cDNA Reverse Transcription Kit (Applied Biosystems, California, USA). RT-PCR cycles consisted in 10′ 25°C, 2 hours at 37°C, and 5′ at 85°C. The cDNA was analyzed by quantitative real-time PCR using Brilliant III Ultra-Fast SYBR Green QPCR Master Mix (Agilent Technologies, La Jolla, CA) and the Mx3005P sequence detector System (Stratagene). Q-PCR reaction consisted of 40 cycles at 94°C (15 sec), then 60°C (30 sec) and 72°C (30 sec). Endogenous *WAS* expression was assessed using the following primers: forward, 5′-AGGCTGTGCGGCAGGAGAT-3′ and reverse 5′-CAGTGGACCAGAACGACCCTTG-3′), forward GCTCTGGGAACAGGAGCTG and reverse CTCGTCCTCGTCTGCAAAGT. The *GAPDH* gene was used as control and relative expression was calculated using de ΔCT method [43]. GAPDH primers were: forward, 5′-GAAGGTGAAGGTCGGAGTC-3′ and reverse, 5′-GAAGATGGTGATGGGATTTC-3′.

### Neural Differentiation of hESCs

The protocol was slightly modified from that described by Pankratz [44]. Briefly, hESC lines were grown in suspension as hEBs in hESCs mdium as described previously for 4 days. The hEBs were then cultured in neural medium composed by DMEM/F12, nonessential amino acids, 2 µg/ml heparin, and the neural cell supplement N2 (Gibco) for 3 additional days. Early neural differentiation was evaluated at day 8 of culture by dissociating and staining hEB single cells with the mouse anti-human A2B5 (a neural embryonic antigen marker) (1∶25 dilution; Miltenyi Biotech) or the corresponding isotype control. APC-conjugated goat anti-mouse antibody was used as secondary antibody (Miltenyi Biotech).

### Endothelial Differentiation of hESCs

To promote endothelial differentiation, the different hESCs lines were seeded at density 10–30×10^3^ cells/cm^2^ on plates coated with 0.1% gelatin in EGM-2 complete media (Lonza Walkersville). 12 days later, the cells were detached with TryPLE (Invitrogen) and washed with PBS buffer supplemented with 2 mM EDTA and 3% FBS. Endothelial differentiation was determined by FACS analysis incubating with mouse anti-human VE-Cadherine-PE (eBiosciences) using mouse IgG-PE isotype as control. Live cells identified by 7-AAD viability dye exclusion were analyzed for CD105, VE-cadherine and eGFP expression using a FACS Canto II flow cytometer equipped with the FACS Diva analysis software (BD Biosciences).

### Statistical Analysis

All data are expressed as mean ± SEM. Statistical comparisons were performed (GraphPad Prism program) with nonparametric test (Mann-Whitney test), two-tailed P value (95% confidence interval). Statistical significance was defined as a *P* value <0.05.

## Results

### Endogenous *WAS* Gene Expression Parallels Hematopoietic Commitment from hESCs

**Figure 1:**
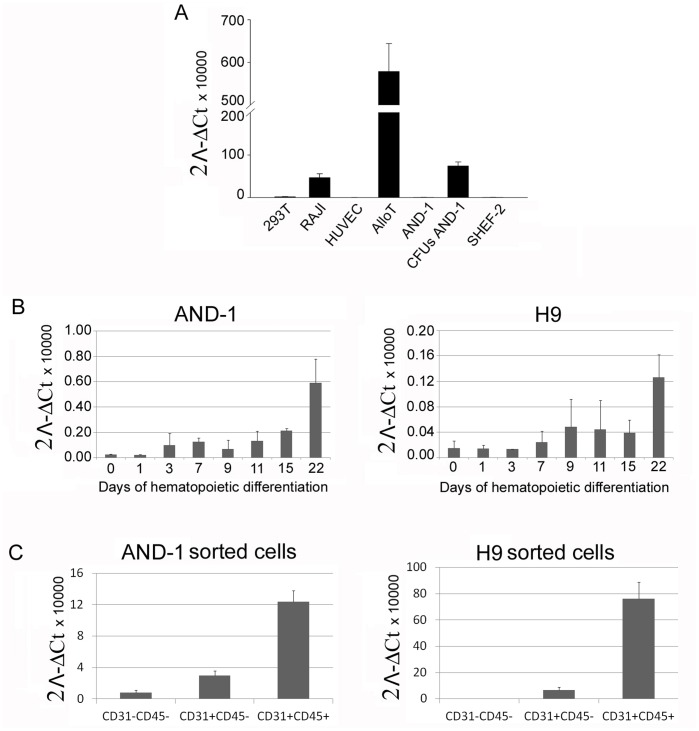
*WAS* gene expression is restricted to hematopoietic cells and hemogenic progenitors. (**A**) Analysis of *WAS* gene expression in different cell lines. mRNA was obtained from hematopoietic cell lines (AlloT and Raji), hESCs-derived myeloid CFUs (And-1 CFUs), undifferentiated hESCs (AND-1 and SHEF-2), a human fibroblastic cell line (293T) and human endothelial cells (HUVEC) and analyzed by RT-PCR for *WAS* expression (see M&M for details). (**B**) Time-course analysis of *WAS* gene expression during hematopoietic differentiation of AND-1 and H9 hESCs (see M&M and Figure S1 for details). mRNA was extracted at different days during differentiation as indicated and *WAS* expression analyzed by RT-PCR. (**C**) Analysis of *WAS* gene expression in hemogenic progenitors and hematopoietic cells. CD45^−^CD31^+^ (containing hemogenic progenitors) and CD45^+^CD31^+^ (containing hematopoietic cells) populations were sorter from AND-1 (left) and H9 (right) hESCs after 15 days of hematopoietic differentiation. *WAS* mRNA relative levels were determined in sorted cells by RT-PCR. ΔCt value for *WAS* expression was obtained using GAPDH as reference gene in all experiments. CFUs: Colony Forming Units.

Although the hematopoietic-specific expression of *WAS* gene is well established [45], [46], [47], its expression pattern during early hematopoietic development remains unknown. We therefore studied *WAS* gene expression in hESCs during hematopoietic differentiation (Figure 1). We first analyzed by RT-PCR the relative endogenous *WAS* expression in non-hematopoietic cells (293T and HUVEC) and hematopoietic (AlloT and Raji) cell lines as well as in undifferentiated hESCs (AND-1 and SHEF-2) and hESCs-derivedCFUs from AND-1 (Figure 1A). We confirmed that only hematopoietic cell lines (AlloT and Raji) or hESCs-derived hematopoietic cells (AND-1 CFUs) expressed *WAS* over background levels (Figure 1A). We could not detect any significant expression of *WAS* in endothelial cells (HUVEC), nor in undifferentiated hESCs (AND-1 and SHEF-2). However, upon hematopoietic differentiation (see Figure S1 for details), *WAS* expression appears early (day 3–5) and increased over time (Figure 1B), paralleling hematopoietic commitment of hESCs. In order to further characterize *WAS* expression during hematopoietic commitment, we sorted CD45**^−^** CD31**^−^** non-hematopoietic cells, CD45**^−^**CD31^+^ hemogenic progenitors and CD45^+^CD31^+^ hematopoietic cells at day 15 of differentiation and analyzed for *WAS* expression (Figure 1C). In line with the expression kinetics shown in Figure 1B, CD45^+^ hematopoietic cells contained the highest levels of *WAS* mRNA followed by CD31^+^CD45^−^ hemogenic progenitors. Together, this data indicates that WAS expression parallels hematopoietic commitment from hESCs.

### Expression of *WAS*-promoter Driven LVs is Restricted to hESCs-derived Hematopoietic Lineage

**Figure 2:**
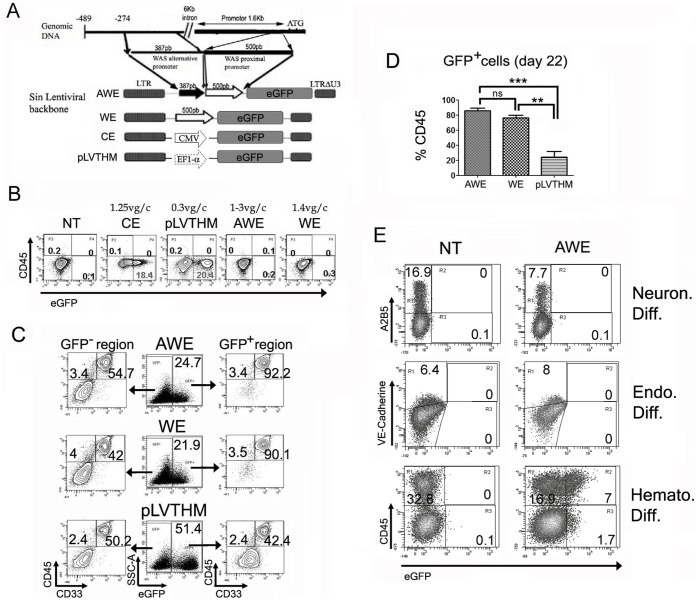
Specific expression of *WAS*-promoter driven LVs in hESCs-derived hematopoietic cells. (**A**) Representation of the SIN LVs used to transduce hESCs. WE vector [31] contains a 500-bp fragment of the human *WAS* proximal promoter driving the expression of the reporter gene eGFP. The AWE vector [33] contains an aditional 387-bp fragment of the *WAS* alternative promoter. The CE and pLVTHM are control lentiviral vectors expressing constitutively eGFP through the CMV and the EF1α promoters, respectively. (**B**) Constitutive LVs (CE and pLVTHM) express eGFP in hESC undifferentiated cells (AND-1) while the hematopoietic-specific AWE and WE LVs are silent. AND-1 cells were transduced with the LVs in order to obtain 0.5–3 vg/c (see materials and methods). (**C**) Hematopoietic differentiation induces eGFP expression in AWE and WE-transduced hESCs. AWE-, WE- and pLVTHM- transduced H9 cells were prompt to differentiate and 22 days later analyzed for eGFP expression (Middle plots). eGFP^+^ (right) and eGFP^−^ (left) populations were analyzed for expression of mature hematopoietic markers CD45 and CD33. Note that WE and AWE-transduced cells (top and middle plots) mark specifically CD45^+^CD33^+^ cells while pLVTHM (bottom plots) express eGFP equally in hematopoietic and non-hematopoietic cells. (**D**) Graph showing the percentage of CD45^+^ cells within the eGFP^+^ population from AWE-, WE- and pLVTHM-transduced hESCs at day 22. AWE and WE- transduced cells did not show statistics differences. pLVTHM-transduced hESCs were used as controls. Data are average of at least three independent experiments +/− SEM *** P = 0.0002; ** P = 0.0023 (**E**) Neuronal and endothelial differentiation of AWE-transduced hESCs does not induce eGFP expression. AWE-transduced H9 cells (AWE) were differentiated into neural progenitors (Top plots) and endothelium (middle plots) and analyzed for eGFP, A2B5 (an early neuroectodermal marker) and VE-Cadherine (an endothelial marker) expression. The AWE-transduced H9 cells were also used for EB-mediated hematopoietic differentiation and analyzed after 22 days for eGFP and CD45 (Right plot). Untransduced (NT) H9 were used as a negative control for eGFP expression.

We have previously described the development of two hematopoietic-specific *WAS*-promoter-driven LVs: WE [31] an AWE [33] (Figure 2A). The WE LV harbours a 500 bp fragment from the *WAS* proximal promoter driving the expression of eGFP and the AWE LV contains an additional 387 pb from the WAS alternative promoter upstream of the proximal promoter. In order to determine the ability of WE and AWE LVs to transduce hESCs, we incubated the AND-1 cell line with the LV-containing supernatants. Two other constitutive vectors, CE [40] and pLVTHM [39], driving eGFP expression through the CMV and EF1-α promoters respectively, were used as controls. All LVs efficiently integrated into the hESCs (as determined by Q-PCR) achieving 0.6–1.4 vector genome per cell (vg/c). However, only the constitutive vectors CE and pLVTHM expressed the transgene in the undifferentiated hESCs (Figure 2B). The transduction protocol did not affect the pluripotency of the hESCs as demonstrated by the expression of pluripotency markers and their ability to form teratomas in NOD-SCID mice (data not shown).

We next studied whether the WE and AWE-transduced hESCs expressed eGFP after hematopoietic differentiation. Transduced hESCs were allowed to differentiate towards the hematopoietic lineage through EB formation in the presence of hematopoietic cytokines and 22 days later we measured the emergence of hematopoietic and eGFP^+^ cells. Upon hematopoietic differentiation, eGFP expression was evident in both *WAS*-promoter driven LVs. Importantly over 95% of the eGFP^+^ cells were CD45^+^ and over 90% were CD45^+^ CD33^+^ myeloid cells (Figure 2C, right plots). This indicates that eGFP^+^ expression is labelling specifically CD45^+^ hematopoietic cells at day 22 of differentiation. However, the constitutive pLVTHM vector expressed eGFP equally in CD45^−^ and CD45^+^ cells (Figure 2C, bottom plots).

To compare the specificity of expression of WE versus AWE vectors we analyzed the percentage of CD45^+^ cells within the eGFP^+^ population at day 22 of differentiation (Figure 2D). Both vectors performed very similar in marking hESCs-derived CD45^+^ cells and we did not detected significance when comparing WE and AWE vector (Figure 2D and Figure S2). However, the AWE vector was slightly better than the WE vector when compared to pLVTHM (P = 0.0002 for AWE and P = 0.0023 for WE). Based on this data and on previous studies showing a more physiologic expression of the AWE vector [33], we decided to focus in the AWE vectors for subsequent experiments.

We further analysed whether differentiation of AWE-transduced-hESCs to a non-hematopoietic lineage promoted transgene expression. We used the AWE-transduced hESCs to differentiate toward neuronal and endothelial lineages. The AWE vector was unable to express eGFP when the hESC was differentiated toward neither of the lineages (Figure 2E, top and middle plots). On the other hand, when these hESCs were differentiated to the hematopoietic lineage, eGFP started to be expressed in the CD45^+^ cells (Figure 2E, bottom plots and Figure 2C).

**Figure 3:**
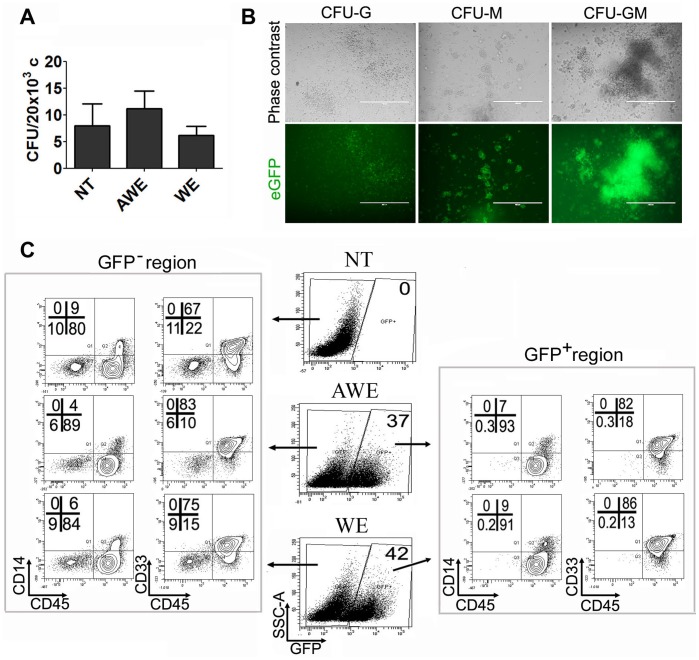
*WAS*-promoter driven LVs efficiently express eGFP in hESC-derived myeloid colonies. AWE and WE-transduced hESCs were plated in EB hematopoietic differentiation media for 15 days and then seeded in methylcellulose H4434 (Stem Cell Technologies, Vancouver, Canada). (**A**) Graph showing CFU efficiency obtained from AWE and WE-transduced hESCs compared to untransduced hESCs (NT). Data are shown as average from three independent experiments +/− SEM. (**B**) Phase contrast (Top panels) and fluorescence (bottom panels) microphotographs from AWE-transduced hESCs derived CFUs. (**C**) Phenotypic analysis of cells derived from AWE and WE-transduced hESCs. eGFP^+^ (right plots) and eGFP^−^ (left plots) populations were analyzed for expression of mature hematopoietic markers CD45, CD33 and CD14.

To further characterize AWE and WE expression pattern we plated transduced-hESCs at day 15 of differentiation on methylcellulose. CFU hematopoietic colonies were scored and analyzed for CD45, CD33 and CD14 hematopoietic markers (Figure 3). The results showed that the transduction protocol did not affect CFU potential of the hESCs-derived progenitors (Figure 3A) and about 40% of the colonies expressed eGFP regardless of the colony subtype (Figure 3B, Figure S3 and data not shown). All eGFP^+^ cells were CD45^+^ (Figure 3C, right plots), about 82–86% CD45^+^CD33^+^ and 7–9% CD45^+^CD14^+^. As expected, neither the WE nor the AWE LVs expressed eGFP in the remaining CD45^−^ cells (Figure 3C).

### The AWE Vectors Mark CD45^−^CD31^low/−^CD34^−^, CD45^−^CD31^+^ and CD45^+^CD34^+^ Cells Emerging from hESCs at Early Days of Hematopoietic Differentiation

**Figure 4:**
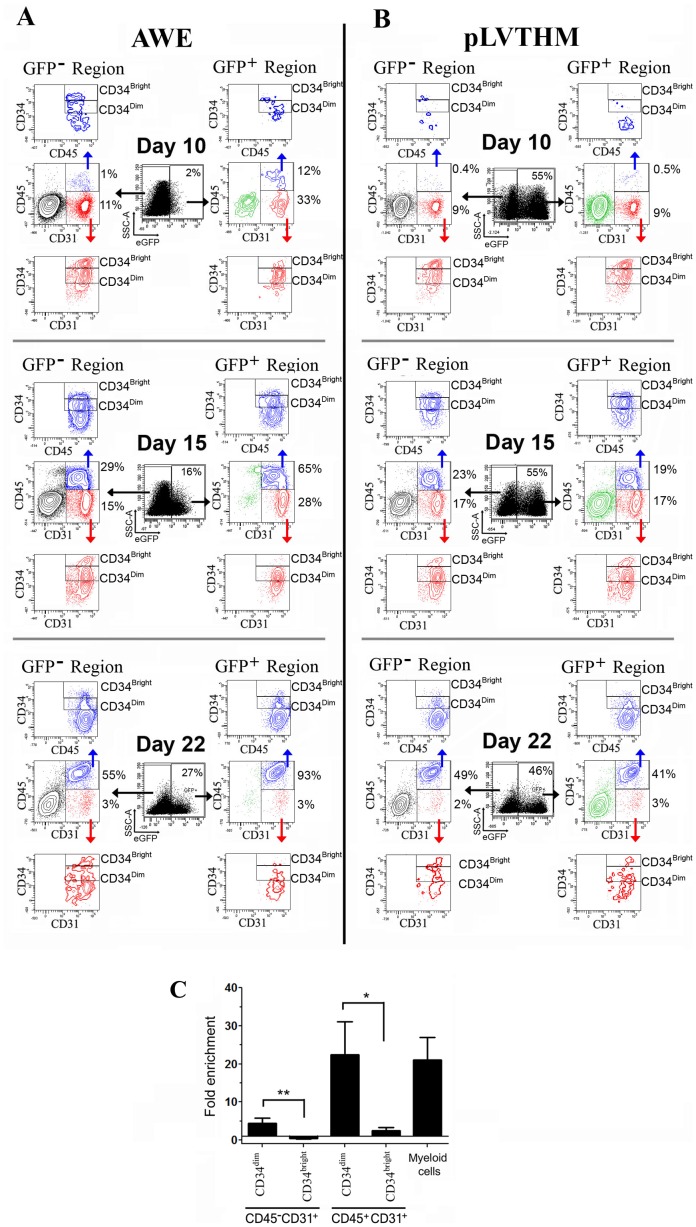
The AWE vectors identify different sub-populations at different times of hematopoietic differentiations. (**A**) AWE-transduced H9 cells were incubated in hematopoietic differentiation media and analyzed for CD45, CD31 and CD34 expression at days 10, 15 and 22. eGFP^+^ (right) and eGFP^−^ (left) populations were first analyzed for expression of CD45 and CD31. hESCs CD31^+^CD45^+^ and CD31^+^CD45^−^ were further analyzed for expression of CD34 (top and bottom plots respectively). (**B**) A similar experiment as in A) was performed using pLVTHM -transduced H9 cells. Note that using the pLVTHM constitutive vectors there are no differences in eGFP expression from day 10 to day 22. (**C**) AWE- marked cells are enriched in CD34^dim^ hemogenic progenitors and hematopoietic cells. Graph representing fold enrichment of eGFP^+^ versus eGFP^−^ population in hemogenic progenitors (CD45^−^CD31^+^), hematopoietic progenitors (CD45^+^CD34^+^) and myeloid cells (CD45^+^CD33^+^). CD34^dim^ and CD34^bright^ progenitor cells were analyzed independently. Data were obtained by dividing the percentages of the different cell types in the eGFP^+^ fraction of the AWE-transduced hESCs (from Figure 4A, right plots) with the values obtained in the eGFP^−^ population (Figure 4A, left plots) and plotted as fold enrichment. Data represent average of at least five separate experiments (+/− SEM) using H9 and AND-1cells at medium stage of hematopoietic development (day 10 for H9 and day 15 for AND-1). ** P = 0.008; * P = 0.032.

We further investigated the eGFP expression kinetics through hematopoietic differentiation of AWE- and pLVTHM-transduced-hESCs. Transduced hESCs were allowed to differentiate towards the hematopoietic lineage and analysed at different time points (days 10, 15 and 22). eGFP expression driven by the AWE LV was manifested at day 10 and increased progressively at days 15 and 22 (Figure 4A, black plots) paralleling CD45 expression (Figure 4A and Figure S4). The phenotypic analysis of the eGFP^+^ cells showed specific marking of different subpopulations at different days of differentiation. At day 10, around 33% of the eGFP^+^ cells were CD45^−^CD31^+^CD34^+^, markers characteristic of hemogenic progenitors (Figure 4A, Day 10, right plots, highlighted in red). 12% were CD45^+^CD31^+^ hematopoietic cells of which 60% were CD34^+^, markers that identify hematopoietic precursors (Figure 4A, Day 10, right plots, highlighted in blue). The remaining 55% eGFP^+^ cells were CD45^−^CD31^low/−^ (Figure 4A, Day 10, right plots, highlighted in green). Note that the CD31 expression levels of eGFP^+^ cells is slightly higher than the observed in eGFP^−^ cells (Figure 4A, right plots, green population, versus left plots black population; day 10 and day 15). Further analysis of this population showed that they were also negative for CD34 (Figure S5). We therefore annotate this population as CD45^−^CD31^low/−^CD34^−^. As differentiation continued to days 15 and 22, most of the eGFP^+^CD45^−^ populations disappeared (Figure 4A, middle-right plots, highlighted in green and red) and over 93% of the eGFP^+^ cells became CD45^+^CD31^+^. The phenotype of pLVTHM-transduced hESCs was identical in the eGFP^+^ and eGFP^−^ regions (Figure 4B), indicating equal expression of this vector in all populations.

Interestingly, the AWE LV marked only CD31^+^ cells expressing moderate-low levels of CD34 (CD34^dim^). This is more evident in the CD45^−^CD31^+^ population at day 10 of differentiation where the CD34^bright^ population in the eGFP^-^ region completely disappeared in the eGFP^+^ region (Figure 4A, highlighted in red, Figure 4C and Figure S5). The same was observed for the WE LV (Figure S6). To confirm these data we used two different hESCs lines (H9 and AND-1) and compared the fold enrichment in CD34^dim^ and CD34^bright^ subpopulations from hemogenic (CD45^−^CD31^+^) and hematopoietic progenitors (CD45^+^CD31^+^) in the eGFP^+^ versus eGFP^−^ populations (Figure 4C). We also included the CD45^+^CD33^+^CD34^−^ cells in this study to compare the fold enrichment in progenitors versus hematopoietic mature cells. The data corroborated that eGFP^+^ cells specifically tag CD34^dim^ cells in both CD45^−^CD31^+^ and CD45^+^CD31^+^ cells.

### The AWE Vectors Mark Early Hemogenic Progenitors

**Figure 5:**
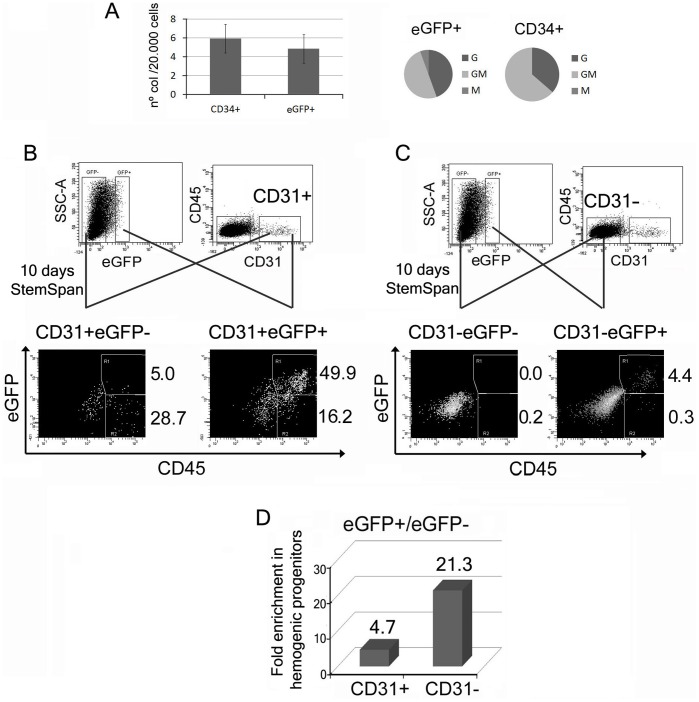
The AWE vector tag hemogenic and hematopoietic progenitors. (**A**) CFU potential of eGFP+ cells. AWE- transduced H9 cells were incubated in EB hematopoietic differentiation media. 10–12 days later, eGFP^+^ and CD34^+^ cells were sorted and seeded in methylcellulose H4434 (Stem Cell Technologies, Vancouver, Canada). Left graph shows CFU efficiency obtained from eGFP+ sorted cells compared to CD34^+^. Data are shown as average from three independent experiments +/− SEM. Right graphs shows relative frequency of the different types of colonies (G =  granulocytes; GM =  Granulocytes/macrophages; M = Macrophages) obtained from eGFP^+^ (left) and CD34^+^ (right) sorted cells. (**B**) Hemogenic potential of CD45^−^CD31^+^ cells expressing eGFP. AWE- transduced H9 cells were incubated in EB hematopoietic differentiation media. 10–12 days later the cells were analyzed for eGFP (top left plot) as well as CD45 and CD31 expression (top right plot). CD31^+^eGFP^−^ (left panels) and CD31^+^eGFP^+^ (right panels) were sorted and induced in StemSpan with Flt-3L, SCF, IL-3, IL-6 and G-CSF(see M&M). Expression of CD45 and eGFP was analyzed 10 days later (bottom-black plots). (**C**) Hemogenic potential of CD45^−^CD31^−^ cells expressing eGFP. AWE- transduced H9 cells were incubated in EB hematopoietic differentiation media. 10–12 days later differentiated cells were analyzed as above. CD31^−^eGFP^−^ (left panels) and CD31^−^eGFP^+^ (right panels) were sorted and induced to differentiate toward haematopoiesis as above. Note that the CD45^−^CD31^−^eGFP^+^ cells (but not the CD45^−^CD31^−^eGFP^−^) generate CD45^+^ cells. (**D**) Hemogenic efficiency of AWE-labelled CD45^−^ populations. Graph showing the fold enrichment in hemogenic progenitors of the eGFP^+^ versus the eGFP^−^ cells on CD31^+^ and CD31^−^ cells. Data were obtained by dividing the percentages of CD45^+^ cells derived from the eGFP^+^CD31^+^ sorted cells with the values obtained with the eGFP^−^CD31^+^ sorted cells (left column) or eGFP^+^CD31^−^ sorted cells with eGFP^−^CD31^−^ sorted cells (right column).

We have showed that eGFP^+^ cells emerging from differentiated AWE-transduced hESCs at day 10 expressed different markers identifying hemogenic (CD45^−^CD31^+^CD34^dim^) and hematopoietic progenitors (CD45^+^CD31^+^CD34^dim^) as well as a singular CD45^−^CD31^dim/−^CD34^−^ population. Our next aim was therefore to determine whether these eGFP^+^ populations had real hemogenic potential. In first place we sorted eGFP^+^ as well as CD34^+^ cells from hematopoietic differentiated hESCs (day 10) and analyzed its CFU potential in methylcellulose (Figure 5A). We showed that the eGFP^+^ cells produced a similar number of hematopoietic colonies than CD34^+^ cells confirming the ability of the AWE vector to tag hematopoietic precursors.

We next investigated whether the eGFP^+^ cells identify a distinct population inside the CD45^−^CD31^+^ since they differ in the expression levels of CD34. We separated CD45^−^CD31^+^eGFP^−^ (CD34^bright^ and CD34^dim^) and CD45^−^CD31^+^eGFP^+^ (CD34^dim^) from day 10 hESCs and incubated them on StemSpan media supplemented with cytokines (Figure 5B). Interestingly the data showed that the eGFP^+^CD45^−^CD31^+^ population generate 5 times more CD45^+^ cells than the eGFP^−^CD45^−^CD31^+^ cells (Figures 5B and 5D). This indicates that the AWE vector can mark a subpopulation inside the CD45^−^CD31^+^ cells with higher hemogenic potential, probably due to the enrichment in hematopoietic-restricted (CD34^dim^) progenitors.

The expression of eGFP in the CD45^−^CD31^low/−^CD34^−^ hESCs-derived cells prompt us to investigate whether these cells represented a hemogenic precursor. We sorted CD45^−^CD31^−^eGFP^−^ and CD45^−^CD31^−^eGFP^+^ (see Figure 5C). Post-sorting analysis of both populations showed a slightly higher expression of CD31 on eGFP^+^ cells compared to eGFP^−^ cells, indicating that the eGFP^+^ express low but significant amounts of CD31 (Figure S7). Both populations (CD45^−^CD31^low/−^eGFP^+^ and CD45^−^CD31^−^eGFP^−^) were incubated in StemSpan media supplemented by hematopoietic factors (See M&M). Interestingly, only the CD45^−^CD31^low/−^eGFP^+^ sorted cells render CD45 positive cells that were also eGFP^+^ (Figure 5C, right-black plots) indicating that the AWE vector is able to identify a subpopulation, CD45^−^CD31^low/−^CD34^−^ with hemogenic potential.

## Discussion

Tracing lineage specification demands the ability to efficiently express a reporter/marker gene (i.e. *eGFP*) exclusively when lineage-specific cells emerge *in vitro*/*in vivo*. In the present manuscript we have studied the feasibility of specifically marking hESC-derived hematopoietic cells with *WAS*-promoter driven lentiviral vectors (WE and AWE). We were also interested in determining the ability of both vectors to mimic the expression pattern of the endogenous *WAS* gene during the different stages of human hematopoietic differentiation. We have shown that hESCs do not express the *WAS* gene until they are committed to hematopoietic lineage. *WAS* expression started to be detected in hemogenic progenitor cells (CD45^−^CD31^+^) and to a higher extent in the CD45^+^ hematopoietic cells. This is the first report showing that the *WAS* gene is expressed in human hemogenic progenitors.

Several groups have developed cardiac-specific LVs able to specifically mark cardiomyogenic cells derived from hESCs [16], [17], [18]. The establishment of transgenic hESCs lines that specifically mark human differentiated cardiomyocytes has allowed the purification (up to 96% pure) and analysis of hESC-derived cardiomyocytes [48]. The use of these transgenic cell lines has also allowed the *in vivo* study of hESCs-derived cardiomyogenic differentiation from hESCs to multiple cardiomyocytes subtypes and the identification of early myocardial precursors derived from hESCs (hMPs) using an alpha-myosin heavy chain (alphaMHC)-GFP reporter line [18]. Since we found that *WAS* gene expression was turned on after hematopoietic differentiation, we hypothesized that a gene delivery vector expressing eGFP through the *WAS* gene promoter could be used to trace hematopoietic cells derived from hESCs. Our experiments showed that eGFP expression in AWE and WE-transduced hESCs followed a similar expression pattern than the endogenous *WAS* gene during hematopoietic differentiation. Although both LVs were very efficient integrating their cargo into the hESCs they failed to express the transgene in undifferentiated hESCs and required hematopoietic differentiation to express eGFP. This data indicates that undifferentiated hESC do not contain the required transcription factors to activate the *WAS* promoter, as suggested also by the absence of endogenous *WAS* mRNA in these cells. Although the expression pattern of the WE and AWE vectors were very similar, we detected a slightly higher (although not significant) specificity of the AWE. In addition, in previous studies [33] we showed a more physiological behaviour of the AWE vectors. We therefore selected the AWE vector for in-detail analysis of the WAS-promoter driven LVs as a tool to identify hemogenic progenitors and hematopoietic cells emerging from hESCs.

We first showed that hESCs were not affected in their potential to differentiate to the different germ layers after LVs transduction andthe hematopoietic derivatives from transduced-hESCs were phenotypically indistinguishable to control hESCs. A detailed analysis of the eGFP expressing cells emerging during hematopoietic differentiation of the AWE-transduced hESCs showed that this vector were able to mark different cell types. At early stages of hematopoietic differentiation, the AWE vector mark cells previously identified as hemogenic (CD45^−^CD31^+^CD34^+^) and hematopoietic progenitors (CD45^+^CD34^+^) as well as CD45^−^CD31^low/−^CD34^−^ cells. We demonstrated that WAS-promoter driven LVs identify hematopoieitic progenitors emerging from hESCs with similar efficiency than selection with anti-CD34 antibodies. Indeed, day10-eGFP expressing cells had similar CFU potential in methylcellulose than CD34^+^ cells isolated from the same culture. These results opened the possibility of isolate progenitors using only eGFP expression or combining eGFP and other known markers for hemogenic and hematopoietic progenitors.

Taken advantage of this possibility we decided to analyze the hemogenic potential of the CD45^−^CD31^+^ and CD45^−^CD31^−^ cells identified by the AWE vectors. An interesting finding in this work is that the AWE mark a subpopulation inside the CD45^−^CD31^+^ cells expressing low-moderate levels of CD34 (CD34^dim^), a characteristic defined for some authors as hematopoietic-restricted progenitors, in contrast to the CD34^bright^ found in hemato-endothelial progenitors [4], [27]. Our experiments showed that the eGFP^+^CD45^−^CD31^+^ cells were up to 6 times better generating CD45^+^ cells than the eGFP^−^CD45^−^CD31^+^ cells when incubated in a medium that allows hematopoietic differentiation. These data clearly demonstrate the hemogenic potential of the eGFP^+^CD45^−^CD31^+^ cells and favour the hypothesis that they could represent a hematopoietic-restricted progenitor. Consequently, the eGFP expression of AWE-transduced hESCs at early-intermediate stages of hematopoietic development could be an interesting marker to study the CD34^dim^ and CD34^bright^ populations. For example, by sorting eGFP^−^CD34^bright^ and eGFP^−^CD34^−^ cells at day 9–10 would be possible to trace the origin of the eGFP^+^CD45^−^CD31^+^CD34^dim^ and eGFP^+^CD45^+^CD31^+^CD34^dim^ cells.

As mentioned before, half of the eGFP^+^ cells at day 10 were negative for the CD45, CD31 and CD34 markers. Since they expressed eGFP through the AWE LVs, they could represent pre-hematopoietic mesodermal precursors able to differentiate into hematopoietic cells. However, another possible explanation would be that the AWE vector is expressing eGFP in non-hematopoietic cells. There were several facts favouring the hypothesis that these eGFP^+^CD45^−^CD31^low/−^CD34^−^ cells could represent a new subpopulation of hemogenic progenitors: a) The AWE eGFP^+^CD45^−^CD31^low/−^CD34^−^ population is transient and only present at early-intermediate stages of the hematopoietic development indicating they could be pre-hemato mesodermal precursors. b) The emergence of CD45^+^ cells coincide with the disappearance of the AWE eGFP^+^CD45^−^CD31^low/−^CD34^−^ cells. c) The eGFP^+^CD45^−^CD31^low/−^CD34^−^ population obtained from the pLVTHM-transduced hESCs do not disappear at later stages of differentiation. In order to corroborate this hypothesis we sorted eGFP^+^CD45^−^CD31^low/−^ and eGFP^−^CD45^−^CD31^−^ cells and showed that only the eGFP^+^ cells have the potential to generate CD45^+^ cells. Therefore the AWE eGFP^+^CD45^−^CD31^low/−^CD34^−^ population represent a previously unidentified hemogenic progenitors. It is interesting to mention a recently published manuscript by the group of Dr Berardi [49] that identified a new adult human circulating CD45^−^Lin^−^CD34^−^CD133^−^ cells that can differentiate to hematopoietic and endothelial cells. These cells are somehow similar to our eGFP^+^CD45^−^CD31^low/−^CD34^−^ population. It would be therefore interesting to further characterize the eGFP^+^CD45^−^CD31^low/−^CD34^−^ population as a possible cellular model mimicking this rare population found in adult blood.

In summary, we have shown the usefulness of *WAS*-promoter driven LVs (AWE and WE) to specifically express transgenes in hESCs-derived hematopoietic cells. We have demonstrated that the AWE and WE LVs specifically expressed eGFP in hemogenic and hematopoietic progenitors at day 10–15 of differentiation. Interestingly, these LVs were able to discriminate between the hematopoietic-restricted hemogenic progenitors (eGFP^+^CD45^−^CD31^+^CD34^dim^) and the hemato-endothelial hemogenic progenitors (eGFP^−^CD45^−^CD31^+^CD34^bright^). Therefore, the characteristic expression pattern of the AWE vector opens up new opportunities to study hematopoietic development. The AWE vector could be used as a tool for isolation/purification of early hematopoietic progenitors, for high throughput screening to identify molecules involved in hematopoiesis and/or for the specific expression of transgenes in hESC-derived hematopoietic cells. In addition our data point to the potential of the *WAS* gene as a new marker for hemogenic and hematopoietic progenitors.

## Supporting Information

Figure S1 **Schematic diagram showing the procedure for hematopoietic differentiation of hESCs. hESC are incubated in non-adherent plates in EBs medium (see M&M for details).** Once the EBs are formed, the media is replaced for EB medium supplemented with hematopoietic cytokines and incubated for 22days. Total RNA was obtained at different the days (0, 1, 3, 5, 7, 11, 15 and 22) during hematopoietic differentiation for RT-PCR. For FACS analysis, EBs were dissociated and analyzed at days 10, 15 and 22. For CFU formation, EBs were dissociated at day 15 and incubated in methylcellulose.
(TIF)

Figure S2 **Lentiviral transduction does not affect hematopoietic differentiation potential of hESCs.** Untransduced nESCs (NT) and AWE- and WE-transduced H9 cells were induced towards hematopoiesis by EBs formation. At day 15 of differentiation (top), the EBs were dissociated and analyzed for CD31 and CD45 expression to determine the percentage of CD31+CD45- hemogenic cells (top graph). At day 22 of differentiation (bottom) we analyzed the percentage of cells expressing CD45. Data represent individual experiments.
(TIF)

Figure S3 ***WAS*** **-promoter driven LVs efficiently express eGFP in hESC-derived myeloid colonies.** Transmission (left panels) and fluorescence (right panels) microphotographs from untransduced (NT), pLVTHM-, AWE- and WE-transduced hESCs. The different hESCs were incubated in EB hematopoietic differentiation media for 15 days and then incubated in methylcellulose H4434 (Stem Cell Technologies, Vancouver, Canada). Pictures were taken after 10 days in methylcellulose. Note the expression of eGFP in both, hematopoietic (colonies, round-shape cells) as well as in non-hematopietic cells (big, adherent cells) in hESCs transduced with the pLVTHM vector. However, the *WAS*-promoter driven LV, AWE and WE, only express eGFP in myeloid colonies and not in background cells.
(TIF)

Figure S4 **Expression pattern of** ***WAS*** **-promoter driven lentiviral vectors parallel CD45 expression during hESCs hematopoietic development.** Graphs showing the percentage of eGFP^+^ (A) and CD45^+^ (B) cells in AWE and WE-transduced H9 cells at different days of hematopoietic differentiation. Untransduced cells (NT) were used as negative controls. Data are average +/−SEM from 3 independent experiments
(TIF)

Figure S5 **Phenotypic analysis of the CD45^−^eGFP^+^cells in AWE-transduced H9 and AND-1 hESCs at days 10–15 of EB differentiation.** pLVTHM- and AWE-transduced H9 and AWE-transduced AND-1 cells were incubated in hematopoietic differentiation media and analyzed for CD45 and eGFP expression (middle plots) after 10 (H9) or 15 (AND-1) days. CD45**^−^**eGFP**^−^** (left plots) and CD45**^−^**eGFP^+^ (right plots) were further analyzed for expression of CD34 and CD31. Compared to the eGFP**^−^** population (left plots) or eGFP^+^ cells from pLVTHM-transduced hESCs (bottom-right plots), eGFP+ cells derived from AWE-transduced hESCs (top and middle right plots) lost most of the CD31^+^CD34^bright^ cells and were enriched in CD31^+^CD34^dim^ cells.
(TIF)

Figure S6 **Phenotypic analysis of eGFP^+^ cells in WE-transduced H9 cells at days 10, 15 and 22 of differentiation.** WE-transduced H9 cells were incubated in hematopoietic differentiation media and analyzed for CD45, CD31 and CD34 expression at days 10, 15 and 22. eGFP^+^ (right) and eGFP**^−^** (left) populations were first analyzed for expression of CD45 and CD31. CD31^+^CD45^+^ and CD31^+^CD45**^−^** were further analyzed for expression of CD34 (top and bottom plots respectively).
(TIF)

Figure S7 **Phenotypoic analysis of sorted populations.** The AWE-transduced H9 cells were induced towards hematopoiesis by EBs formation (see M&M). At day 10 of differentiation, the EBs were dissociated and the different populations sorted. A) Cells were separated based on the expression of eGFP (left plot, arrows) or CD34 (right plot, arrows). eGFP- and eGFP+ sorted cells were analyzed for eGFP expression (left histograms). CD34- and CD34+ cells were analyzed for CD34 expression (right histograms). B) Top plots show the regions used for the sorting of GFP+CD31- and GFP-CD31-. After the separation, the different populations were analyzed for expression of eGFP and CD31 (Bottom histograms). Note the enhanced expression of CD31 in eGFP+ cells compared to the eGFP- cells (bottom histograms)
(TIF)
