# Supplementary figures and images for: Specific Marking of hESCs-Derived Hematopoietic Lineage by WAS-Promoter Driven Lentiviral Vectors
Source: PLoS One. 2012 Jun 14;7(6):e39091. doi: 10.1371/journal.pone.0039091 (PMC3375235; doi:10.1371/journal.pone.0039091)

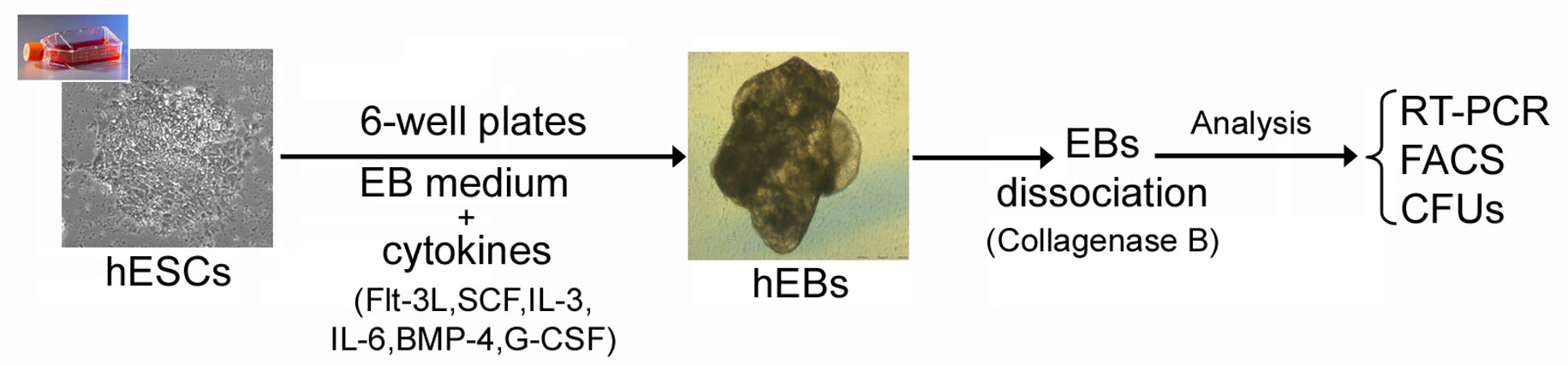

Supplement: Figure S1 — Schematic diagram showing the procedure for hematopoietic differentiation of hESCs. hESC are incubated in non-adherent plates in EBs medium (see M&M for details). Once the EBs are formed, the media is replaced for EB medium supplemented with hematopoietic cytokines and incubated for 22days. Total RNA was obtained at different the days (0, 1, 3, 5, 7, 11, 15 and 22) during hematopoietic differentiation for RT-PCR. For FACS analysis, EBs were dissociated and analyzed at days 10, 15 and 22. For CFU formation, EBs were dissociated at day 15 and incubated in methylcellulose. (TIF) [file pone.0039091.s001.tif]

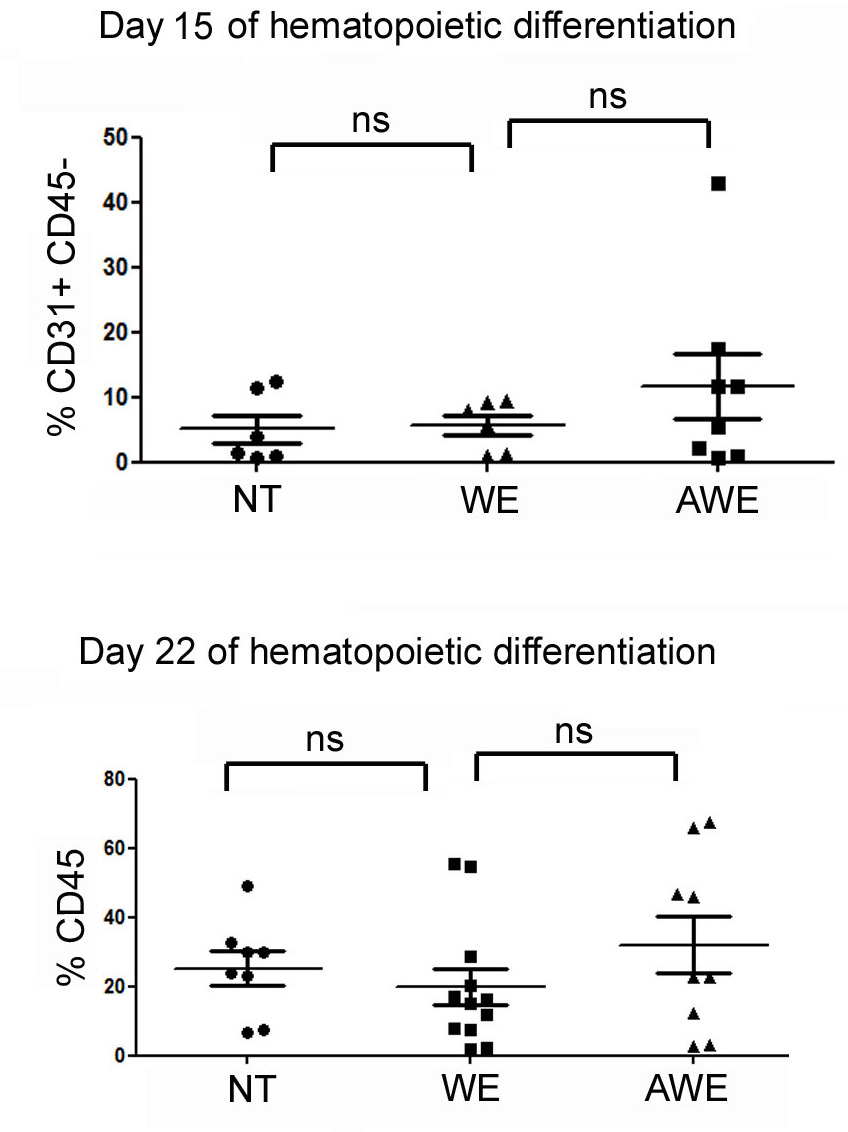

Supplement: Figure S2 — Lentiviral transduction does not affect hematopoietic differentiation potential of hESCs. Untransduced nESCs (NT) and AWE- and WE-transduced H9 cells were induced towards hematopoiesis by EBs formation. At day 15 of differentiation (top), the EBs were dissociated and analyzed for CD31 and CD45 expression to determine the percentage of CD31+CD45- hemogenic cells (top graph). At day 22 of differentiation (bottom) we analyzed the percentage of cells expressing CD45. Data represent individual experiments. (TIF) [file pone.0039091.s002.tif]

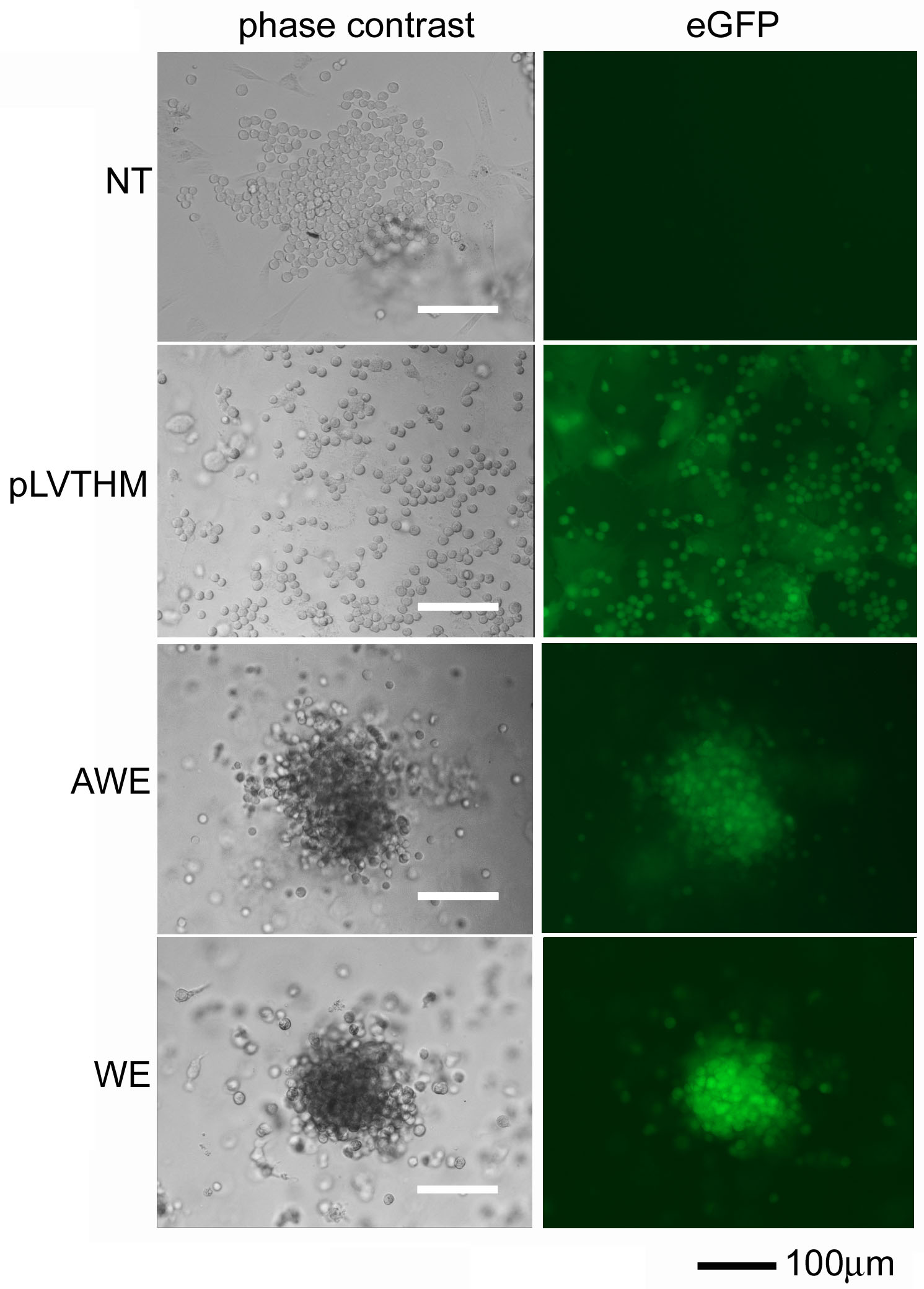

Supplement: Figure S3 — WAS -promoter driven LVs efficiently express eGFP in hESC-derived myeloid colonies. Transmission (left panels) and fluorescence (right panels) microphotographs from untransduced (NT), pLVTHM-, AWE- and WE-transduced hESCs. The different hESCs were incubated in EB hematopoietic differentiation media for 15 days and then incubated in methylcellulose H4434 (Stem Cell Technologies, Vancouver, Canada). Pictures were taken after 10 days in methylcellulose. Note the expression of eGFP in both, hematopoietic (colonies, round-shape cells) as well as in non-hematopietic cells (big, adherent cells) in hESCs transduced with the pLVTHM vector. However, the WAS-promoter driven LV, AWE and WE, only express eGFP in myeloid colonies and not in background cells. (TIF) [file pone.0039091.s003.tif]

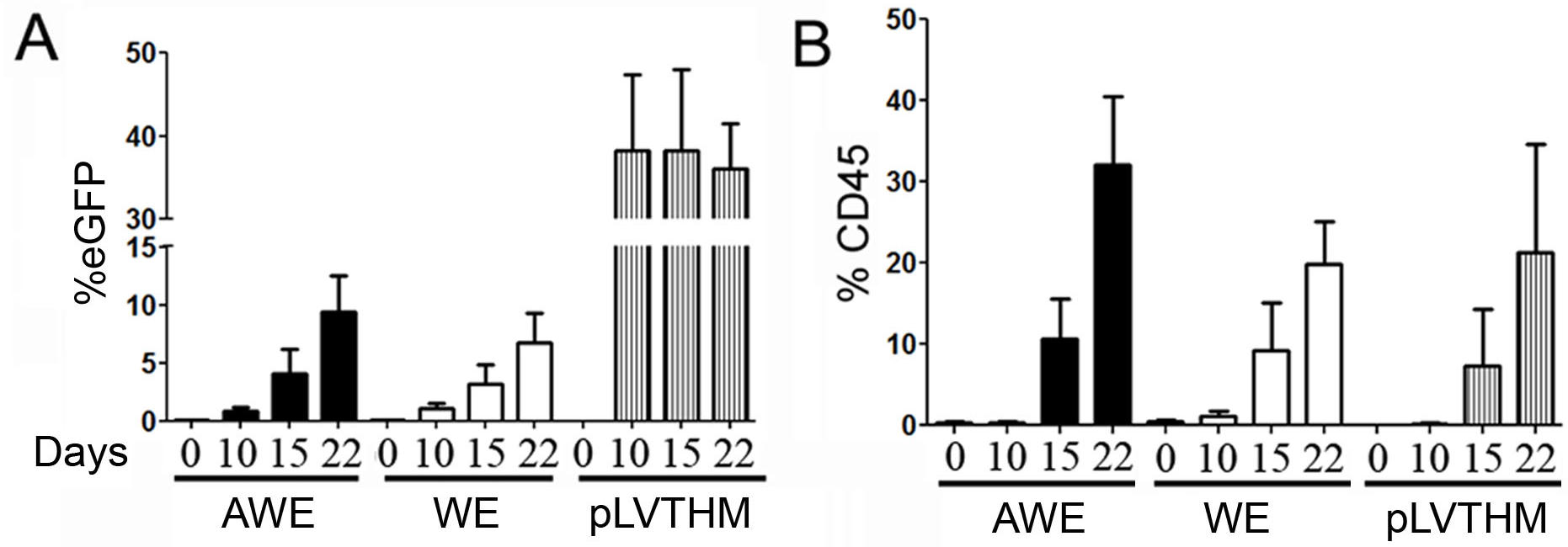

Supplement: Figure S4 — Expression pattern of WAS -promoter driven lentiviral vectors parallel CD45 expression during hESCs hematopoietic development. Graphs showing the percentage of eGFP+ (A) and CD45+ (B) cells in AWE and WE-transduced H9 cells at different days of hematopoietic differentiation. Untransduced cells (NT) were used as negative controls. Data are average +/−SEM from 3 independent experiments (TIF) [file pone.0039091.s004.tif]

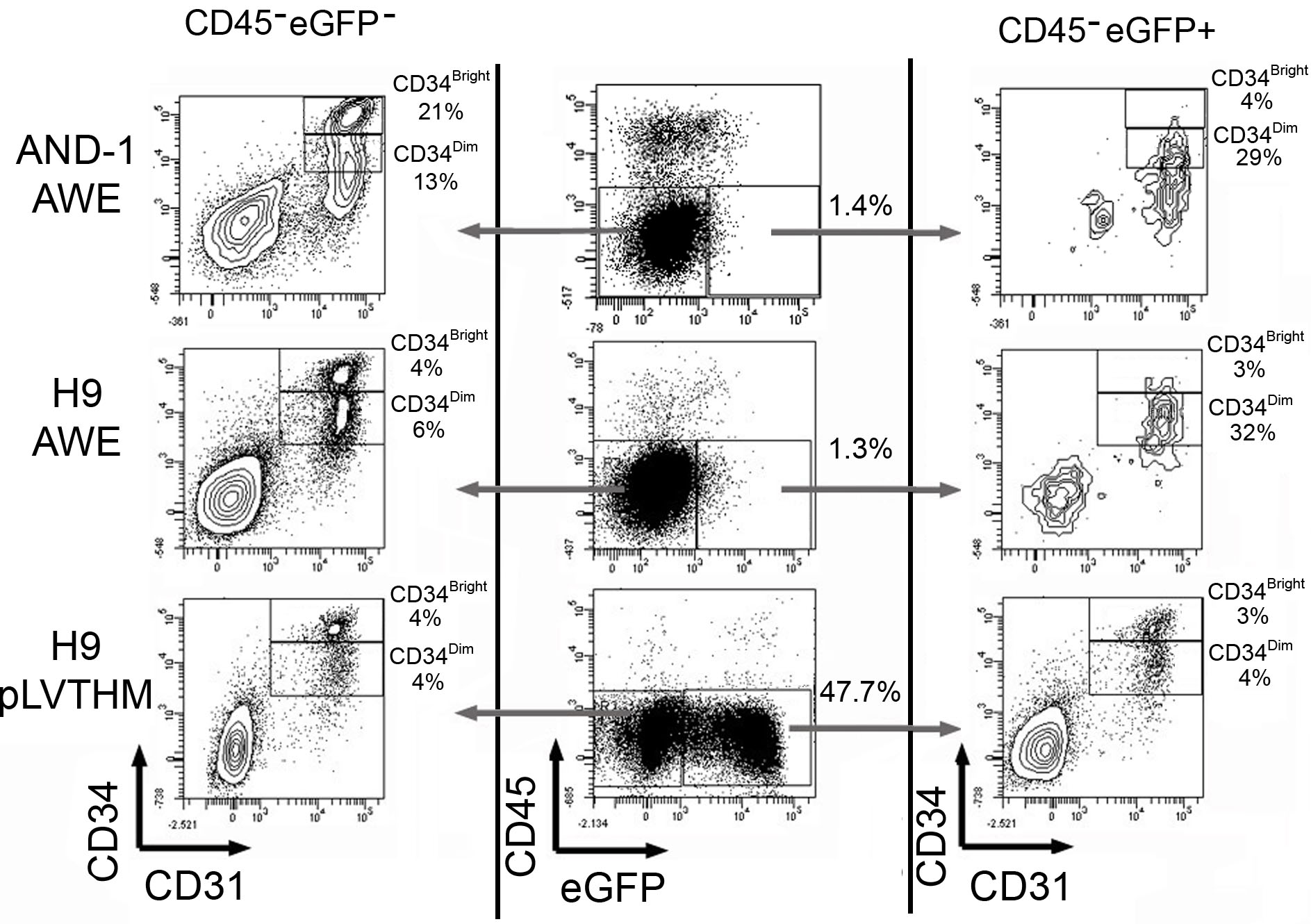

Supplement: Figure S5 — Phenotypic analysis of the CD45−eGFP+cells in AWE-transduced H9 and AND-1 hESCs at days 10–15 of EB differentiation. pLVTHM- and AWE-transduced H9 and AWE-transduced AND-1 cells were incubated in hematopoietic differentiation media and analyzed for CD45 and eGFP expression (middle plots) after 10 (H9) or 15 (AND-1) days. CD45−eGFP− (left plots) and CD45−eGFP+ (right plots) were further analyzed for expression of CD34 and CD31. Compared to the eGFP− population (left plots) or eGFP+ cells from pLVTHM-transduced hESCs (bottom-right plots), eGFP+ cells derived from AWE-transduced hESCs (top and middle right plots) lost most of the CD31+CD34bright cells and were enriched in CD31+CD34dim cells. (TIF) [file pone.0039091.s005.tif]

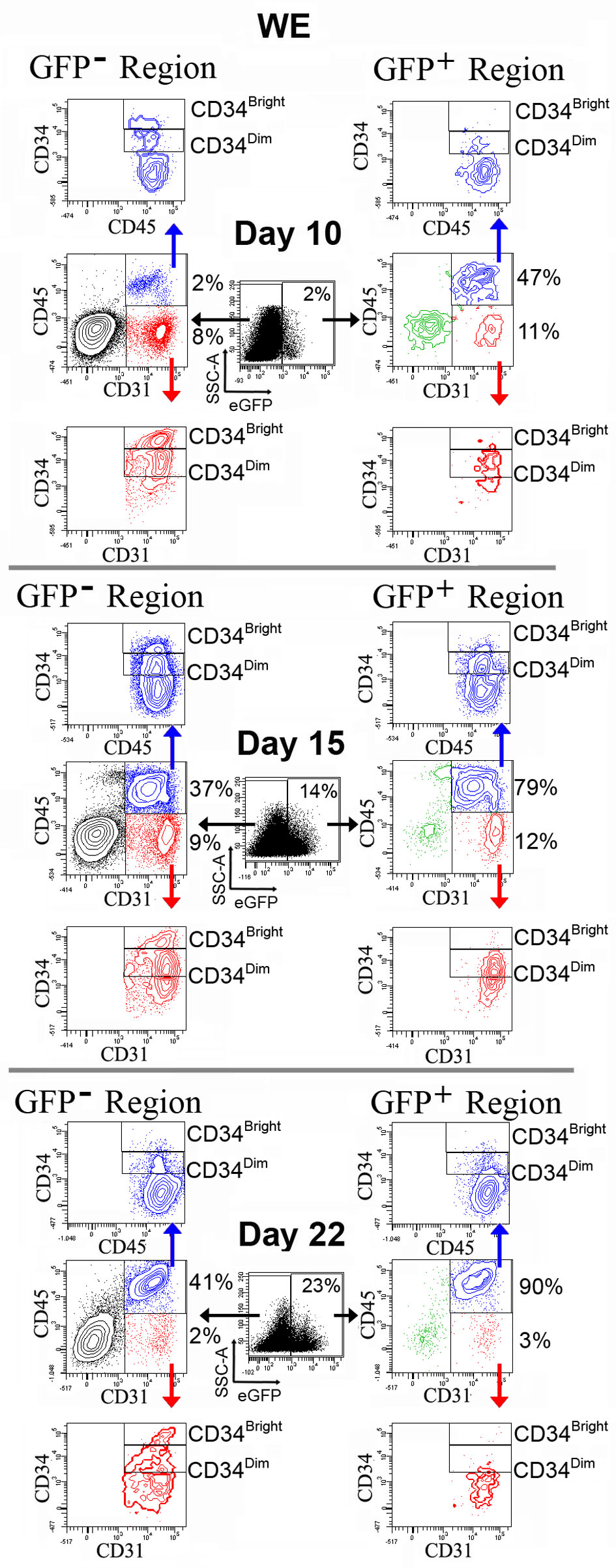

Supplement: Figure S6 — Phenotypic analysis of eGFP+ cells in WE-transduced H9 cells at days 10, 15 and 22 of differentiation. WE-transduced H9 cells were incubated in hematopoietic differentiation media and analyzed for CD45, CD31 and CD34 expression at days 10, 15 and 22. eGFP+ (right) and eGFP− (left) populations were first analyzed for expression of CD45 and CD31. CD31+CD45+ and CD31+CD45− were further analyzed for expression of CD34 (top and bottom plots respectively). (TIF) [file pone.0039091.s006.tif]

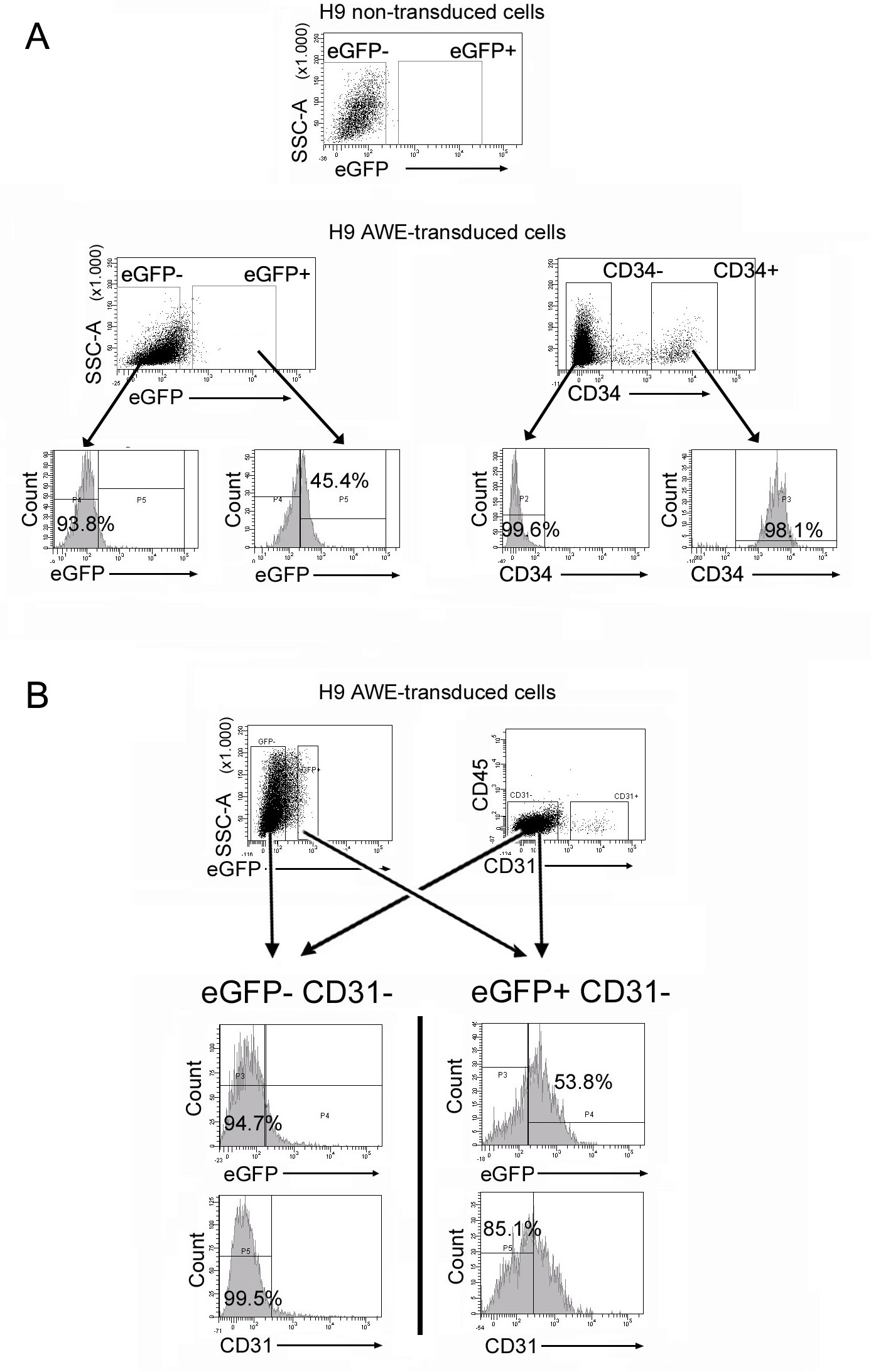

Supplement: Figure S7 — Phenotypoic analysis of sorted populations. The AWE-transduced H9 cells were induced towards hematopoiesis by EBs formation (see M&M). At day 10 of differentiation, the EBs were dissociated and the different populations sorted. A) Cells were separated based on the expression of eGFP (left plot, arrows) or CD34 (right plot, arrows). eGFP- and eGFP+ sorted cells were analyzed for eGFP expression (left histograms). CD34- and CD34+ cells were analyzed for CD34 expression (right histograms). B) Top plots show the regions used for the sorting of GFP+CD31- and GFP-CD31-. After the separation, the different populations were analyzed for expression of eGFP and CD31 (Bottom histograms). Note the enhanced expression of CD31 in eGFP+ cells compared to the eGFP- cells (bottom histograms) (TIF) [file pone.0039091.s007.tif]
